# Evaluation of the Thermal Diffusivity of Carbon/Phenolic Composites (CPCs) through Oxy-Acetylene Torch (OAT) Test—Part 1: Experimental Characterization and Preliminary Validation

**DOI:** 10.3390/polym16050577

**Published:** 2024-02-20

**Authors:** Maurizio Natali, Luigi Torre, Marco Rallini

**Affiliations:** Civil and Environmental Engineering Department, University of Perugia, Strada di Pentima 4, 05100 Terni, Italy; marco.rallini@unipg.it

**Keywords:** thermal protection system (TPS), solid rocket motors (SRMs), carbon/phenolic composites (CPCs), inter laminar shear strength (ILSS), thermal diffusivity

## Abstract

Carbon/Phenolic Composites (CPCs) are essential to manufacture many portions of the nozzle assembly of Solid Rocket Motors (SRMs) which are essential both to preserve the independent access to space as well as for the homeland security. In our research, a feasible approach aimed at preliminary retrieving the in-plane and out-plane thermal diffusivity of CPCs through the Oxy-Acetylene Torch (OAT) tests was validated. The proposed approach showed to be effective and able to bypass some limitations of common protocols, especially in terms of capability to determine the thermal diffusivity of CPCs at high heating rates. A comprehensive work of comparison of the obtained data with state-of-the-art CPCs such as MX-4926 and FM-5014 has also been carried out, evidencing the effectiveness of the proposed method.

## 1. Introduction

During last decades, many Thermal Protection System (TPS) materials have been introduced and optimized for different applications. These materials are used to manufacture propulsion systems such as liquid-fueled rocket engines, Solid Rocket Motors (SRMs)—which are essential to manufacture civilian and military launchers—and, more recently, Hybrid Rocket Motors (HRMs) [1]. They are also used to manufacture the heat shields which protect probes and space vehicles during the hypersonic flight (also known as re-entry) through a planetary atmosphere [1]. Refractory Metals (RMs) and Ultra High Temperature Ceramics (UHTCs) have been evaluated or used as TPS materials. Moreover, carbon-based materials such as graphite, which possesses high heat capacity and high energy of vaporization, are also widely used as TPS material. However, even though graphite has a substantially lower cost and density than RMs and UHTCs, it has the drawbacks of possessing relatively poor mechanical properties and exhibiting problems in terms of resistance to thermal shocks. As a result, carbon fibers [2] are typically introduced in a carbon matrix to produce Carbon/Carbon Composites (CCCs) [1]. CCCs are used in the throat region of SRMs which is exposed to the harshest conditions generated by modern aluminized propellants and where the heat fluxes easily exceed ∼1000 W/cm^2^ [1]. 

Among TPS materials, Polymeric Ablative Materials (PAMs) possess the widest flexibility in terms of properties tunability [1]. Ablation is a self-regulating heat and mass transfer process in which the incident thermal energy is dissipated through a sacrificial material which undergoes endothermic degradation reactions [1]. Carbon/Phenolic Composites (CPCs) have shown the ability to resist heat fluxes up to 30,000 W/cm^2^ [1] and are used to produce heat shields of many civilian and military re-entry objects and many parts of SRM nozzle assemblies while CCCs are preferred in the portion of the motor where the erosive phenomena are more severe such as in the throat region. In fact, using a series of carbonization (up to about 1500 °C), graphitization (up to about 2500 °C) and re-impregnations cycles, CPCs can also be converted into CCCs having the desired density [1]. For all these reasons [2,3], CPCs are virtually the most important class of TPS materials.

In addition to the manufacturing challenges related to CPCs and CCCs reported elsewhere [2,3], another critical phase of the development and qualification of a CPC is related to the evaluation of thermo-physical and thermo-mechanical characterization at the proper heating rates and temperatures. In fact, the strength of PAMs such as CPCs strongly depends on temperature, with mechanical properties rapidly decreasing once the temperature exceeds the glass transition temperature of the polymer [4]. Moreover, the decomposition, the thermo-mechanical stresses and the thermo-physical properties of CPCs are strongly dependent on the type of the hyperthermal environment [1] and intimately related to the heating rate [1,5]. Once tested through proper facilities able to recreate in certain extent the hyperthermal conditions, in which the TPS material must operate [1], analytical models have been introduced to predict the response of the given formulation. 

The necessity for reliable thermo-physical and thermo-mechanical models was recognized in 1960s/1970s when PAMs became essential in most of the high-temperature aerospace applications [4,6,7,8,9,10,11,12,13,14,15,16,17,18,19,20,21,22,23,24,25,26]. The protocols introduced in these researches identified the methods to measure the decomposition kinetic parameters and the thermal properties [8,9,27,28] required for any ablation modeling [1,29,30] through the use of testing techniques such as Differential Scanning Calorimetry (DSC), Differential Thermal Analysis (DTA), Thermo-Gravimetric Analysis (TGA), Thermo-Mechanical Analysis (TMA), Laser Flash Analyzer (LFA), etc.

For TPS materials such as CPCs it is extremely important to evaluate the thermal diffusivity α defined as follows:

(1) $$\alpha =\frac{k}{\rho C_{p}}$$

where k is the thermal conductivity, $C_{p}$ is the heat capacity and ρ is the density of the studied material. 

For PAMs such as CPCs, the density ρ of the given formulation as a function of temperature is usually determined through TGA test up to temperature of 1500/2000 °C and at heating rate from 10 up to about 500 °C/min [1,8,9,27,28].

The possibility to quantify the irreversible heat capacity [9] of TPS materials such as CPCs in a wide range of temperature is essential to properly evaluate the heat of decomposition of the given material. In a previous paper [31], we summarized the efforts to identify a feasible approach able to assess the heat capacity $C_{p}$ as a function of temperature of TPS materials such as CPCs through the use of common DSC and TGA analysis, taking into account the merits and limitations of common DSC/TGA equipments. Accordingly, the heat capacity was calculated through to the following equation:

(2) $$C_{P}\left(T\right)=\frac{1}{m\left(T\right)}\frac{\partial H}{\partial T}=\frac{1}{m\left(T\right)}\frac{\partial H}{\partial t}\frac{\partial t}{\partial T}=\frac{1}{m\left(T\right)}\frac{∆P}{\beta }$$

where ∂H/∂T is the heat flow, $m\left(T\right)$ is the mass of the sample rescaled by the TGA in nitrogen, ∆P is the signal of the DSC (W) and β is the heating rate.

However, most of the papers on the determination of the heat capacity $C_{p}$ of PAMs [10,11,12,13,14,15,16,17,18,19,20,21] were based on very old testing facilities [32] and, in many cases, special testing devices were developed ad hoc [1,5,9]. As an example, Lattimer et al. [9] designed a thermal decomposition apparatus that was able to retrieve the heat capacity of composite materials at high temperature. In order to validate the testing protocols, the authors also carried out a comparison with the DSC results. 

Finally, the thermal conductivity of a material can be evaluated through different protocols as reported in Table 1 [33].

However, most of these protocols are not suitable to fully assess the determination of the thermal conductivity of a PAM such as a CPC especially due to the fact that it is very difficult to impose high heating rates through common electrical heaters. In fact, CPCs used in SRMs commonly experience heating rates in excess of 1000 °C/min and can reach values of 50,000 °C/min [1]. As a result, the main limitation of testing techniques such as the Laser Flash Analyzer (LFA), which are currently used to determine the thermal conductivity of CPCs, is related to the fact that the tests are carried out under steady-state conditions. On the other hand, during the 1960s/1970s, agencies such as NASA used ad hoc designed transient hot-wire equipment to determine the thermal conductivity of CPCs [34,35]. Similarly, Shilav et al. [33,36] developed a test rig aimed at evaluating the thermo-physical properties of Ethylene Propylene Diene Monomer (EPDM) based Elastomeric Heat Shielding Materials (EHSMs) for SRMs. Even though this device is extremely well designed and accurate, its main limitation is related to the heating rate (of about 10 °C/min). 

In general, the methods and experimental devices for the determination of the thermal conductivity and heat capacity of ablative composite materials such as PAMs and CPCs differ from those used for metals. Composites differ from metals in the following ways: (1) they have considerably lower thermal conductivity; (2) there is substantial gas generation in the composite; (3) their thermal properties change non-monotonically; (4) the thermal characteristics of composites depend on rate of heating (or, generally speaking, on ‘history’ of heating) [37].

Moreover, in general, the manufacturing of these apparatuses for the determination of the heat capacity and of the thermal conductivity is not trivial. Certainly, the reports freely available through the NASA Technical Reports Server (NTRS) can help to retrieve some data on International Traffic in Arms Regulations (ITAR) materials (such as the MX-4926 or the FM-5014) and can provide guidance to design special testing apparatus. Nonetheless, also in light of the limitations of most of these protocols, we wanted to validate a simple approach to preliminary evaluate the in-plane and out-plane thermal diffusivity α of PAMs such as CPCs through the Oxy-Acetylene Torch (OAT) tests. From the knowledge of α and of the $C_{p}$ [31], it is possible to retrieve the value of the thermal conductivity k . Using K-type thermocouples to retrieve the in-depth temperatures (as in our experiments), this protocol should be able to measure the thermal diffusivity values from room temperature up to ~1300 °C. This upper limit is also related to the maximum surface temperature that can be typically imposed by an OAT torch to a given ablative material [38,39,40] and (3). Moreover, through the OAT is also possible to reproduce heating rates with values in excess of ~4000 °C/min, enabling the possibility to provide unique insights on the charring phenomena of ablative materials exposed to the harshest conditions [1,41].

In terms of heat transfer analysis and exploitation of the in-depth temperature profiles, the knowledge of the surface thermal boundary conditions in applications such as re-entry flight vehicles or SRM nozzle assemblies is critical to properly design the related TPS as well as for the modeling of the ablation phenomena of charring materials such as CPCs. This is a concern both in the real operational environment of the TPS material as well as in the laboratory test which exploits torches such as the OAT. In fact, harsh ambient conditions related to the given hyperthermal environment [1] or other practical limitations may preclude the use of surface-mounted sensors for measuring the surface temperature and heat flux. To overcome this problem, the temperature profiles obtained from TCs located below the exposed surface can be used to determine the surface thermal boundary conditions. The exploitation of the in-depth temperature data to estimate the surface boundary conditions is known as the Inverse Heat Conduction Problem (IHCP). The diffusion of the thermal front as it penetrates the solid domain—also in the case of charring materials such as CPCs—results in a given temperature profile at the considered depth. Thus, one of the challenges of the IHCP is to exploit the temperature profile at the TC site to estimate the temperature change that occurred at the surface. This implies that small measurement errors in the in-depth TC data are amplified when projected to the surface determining significant errors in the surface prediction. The amplification of measurement errors in the data makes the IHCP prone to errors [1]. Numerous regularization techniques have been developed to obtain a stable prediction of the surface condition. Many methods have been proposed to solve the IHCP. These methods have been summarized in [42].

Some practical applications allow the use of surface-mounted heat-flux gauges. Considerable research has been spent on the development of high-temperature heat-flux gauges that do not require active cooling [42]. However, in many applications, the use of surface-mounted sensors is not practical. In those applications, researchers have proposed the use of plug-type sensors that are instrumented with embedded TCs. For example, plug-type gauges have been used in the Mars Science Laboratory Entry, Decent, and Landing Instrumentation (MEDLI) project [1,43,44]. The MEDLI project used MEDLI Integrated Sensor Plugs (MISPs) that were made of TPS material with four embedded TCs. The measured in-depth TC data were projected in an inverse analysis to obtain an estimation of the surface temperature and heat flux of the MISP. A logical improvement to the use of a plug-type sensor is to perform a calibration test where the sensor is subjected to known surface thermal conditions and the in-depth TC response is measured. This calibration test data, along with in-depth TC data from a new test, can be used to determine the surface thermal conditions that caused the measured in-depth temperature response of the considered test. The Non-Integer System Identification (NISI) method [42] is a system calibration where an impulse function is obtained based on the fractional derivative formulation of the heat equation. The calibration test data are used to determine the calibration coefficients using a least squares method. The surface heat flux of an unknown heating process can be determined using the calibration coefficients along with the in-depth temperature data resulting from the unknown heating process. The Calibration Integral Equation Method (CIEM) is an alternative approach to the concept of system calibration which does not require the calculation of calibration coefficients [42]. 

Myrick et al. [42] presented an experimental investigation of the accuracy of CIEM approach testing an AISI 304 stainless-steel plug-type test sample heated with a laser power source. The one-probe CIEM requires the knowledge of the surface thermal boundary condition of a calibration test along with temperature data from one in-depth probe in order to predict the surface thermal boundary condition of an unknown (reconstruction) heating process given the temperature data from the same probe. Two modified versions of the CIEM that account for temperature-dependent properties were investigated. This study demonstrated that a calibrated plug-type sensor can be used in high temperature applications to accurately reconstruct the surface heat flux and temperature of an unknown surface heating process. The stainless-steel test sample was a proof of concept for a future prototype of this type of calibrated dual measurement gauge. It is worth noting that in a forthcoming work, we will report the effectiveness of the method summarized in this paper to determine the thermal diffusivity of CPCs on AISI 304 stainless-steel cylindrical plugs.

For several decades, Alifanov spent substantial efforts [45,46,47] on the determination of the thermo-physical properties of TPS materials (mostly non ablative) through the design of ad hoc testing facilities and then exploiting IHCP-based algorithms. As an example, in [47] Alifanov presented a work aimed at the verification of the procedure of determining the thermo-physical properties of non-ablative lightweight ceramic TPS materials [1] under representative experimental conditions through the use of the in-depth temperature profiles. The computational algorithm for the exploitation of the experimental data was based on the solution of the inverse heat transfer problem by the method of iteration regularization.

Zhou et al. [48] adopted an IHCP-based approach in a one-dimensional composite slab with rate-dependent pyrolysis chemical reaction and outgassing flow effects using the iterative regularization approach. The thermal properties of the composites were considered to be temperature dependent (like in a CPC and as in this paper). A non-linear conjugate gradient method formulation was developed and applied to solve the IHCP in an organic composite slab whose front-surface was subjected to high intensity periodic laser heating. It was found that for the low-thermal-conductivity composite materials, in addition to the knowledge of the cold-face temperature of the sample, it was necessary to measure the temperatures at an in-depth location as additional information to recover the exposed surface periodic heating condition.

To conclude, the complexity related to the proper evaluation of the thermo-physical and thermo-mechanical properties of TPS materials, as well as the necessity to have good heat transfer (and ablation) models, have been recently recognized both by the European Space Agency (ESA) and NASA in two space programs. In the Vega C program, ESA announced 2 October 2023 the completion of an independent investigation into an anomaly that took place during a static-fire test of a Zefiro 40 motor 28 June 2023. That test was part of efforts to return the Vega C to flight after a December 2022 launch failure blamed on that motor. The test was intended to confirm the performance of the throat insert, which prime contractor Avio had replaced as part of the recommendations into the December 2022 launch failure. That investigation, released in March, concluded that the CCC from the original supplier (Ukrainian company Yuzhnoye), did not meet specifications. ArianeGroup now supplies the throat insert. “The failure of the test is related to the design of the nozzle that was not upgraded with the change in the CCC supplier for the throat insert”, Giovanni Colangelo, ESA’s inspector general said. The geometry of the new throat insert and its different thermo-mechanical properties contributed to the failure [49]. In the Artemis program—in particular in the Artemis 1 test (December 2022)—NASA managers evidenced that during reentry they noted “more erosion” of the PAM used in the heat shield of the vehicles than expected [50]. For this reason, during a media conference on 9 January 2024, the NASA Administrator announced that Artemis 2, the first crewed mission that will send four astronauts around the moon, had been pushed back from the end of 2024 to no earlier than September 2025. Artemis 3, the first crewed landing, was in turn delayed from late 2025 to no earlier than September 2026. The NASA deputy associate administrator for the Moon to Mars Program said the agency was making good progress on understanding what caused the erosion and expected to find a root cause by spring but needed more time to synthesize the data and update models before flying again.

## 2. Materials and Methods

### 2.1. Matrices and Composites

Bakelite PFS121ME, supplied by Hexion (Duisburg, Germany), was selected as a phenolic matrix. This is a liquid phenolic resol with a viscosity of 0.75–1 mPas at 25 °C having methanol as a solvent. The neat CPC formulation referred as C-P was manufactured using the neat PFS121ME. Moreover, a hybrid phenolic-epoxy formulation called P10E was composed by 100 parts of PFS121ME and 10 parts of Epon 828. Epon 828 is an undiluted clear difunctional bisphenol A/epichlorohydrin derived liquid epoxy resin with a viscosity of 100–150 mPas at 25 °C. At the P10E formulation, a stoichiometric amount of hardener TETA (evaluated on the Epon 828 mass) was also added. The resulting CPC was referred as C-P10E. In both cases, a 2 × 2 Twill weave T300-like Polyacrylonitrile (PAN) based on Carbon Fibers (PAN-CFs) fabric (Microtex GG200T1253K with an areal density of 200 g/cm^2^) was used to produce the laminates. The pre-impregnated fabric was cut in suitable shape for being stacked in a mold: 45 layers of prepregs were overlapped to produce each laminate. The application of pressure and temperature allowed to obtain a square shaped (~100 × 100 mm) composite laminate, approximately 9 mm thick. It is worth remarking that all the composite formulations were consolidated at a very low pressure of 1 bar. The curing cycle used was the following: heating from 30 °C to 60 °C @ 2 °C/min and isothermal at 60 °C for 1 h; heating from 60 °C to 80 °C @ 2 °C/min and isothermal at 80 °C for 1 h; heating from 80 °C to 150 °C @ 2 °C/min and isothermal for 30 min. All the details on the manufacturing, characteristics, and mechanical properties of these two composites are reported in [2].

### 2.2. Oxy-Acetylene Torch (OAT) Test

From an experimental point of view, the data (in-depth temperature profiles) that will be exploited to determine the thermal diffusivity of the CPCs were based on the temperature profiles acquired with an OAT [1]. Two CPC formulations were considered in the OAT tests and in our study: the C-P and the C-P10E formulations described in previous section.

**Figure 1:**
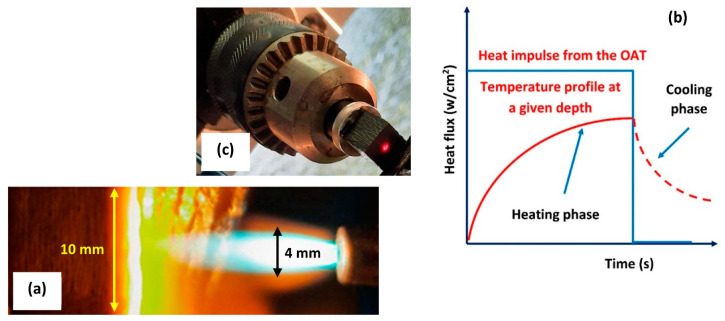
CPC sample and torch tip (side-view) (**a**). The inner core of the torch has an estimated diameter of 4 mm. A typical heat impulse produced by the torch (**b**). Laser pointer of the pyrometer indicating the exact point in which the surface temperature was read (**c**).

In general, the OAT test allows to produce high temperatures (∼3000 °C) and heat fluxes of over 800 W/cm^2^ [51,52,53,54,55,56,57]. Heating rates in excess of 1500 °C/min can also be recreated. In our tests, cubic shaped samples with a size (L) of 10 mm were used. The distance between the sample surface and the flame was set at 15 mm and the heat flux produced by the torch was set at 500 W/cm^2^ (Figure 1a): the calibration of the power of the torch was carried out using a copper slug calorimeter assuming a diameter of the torch plume of ~4 mm. Once the heat flux is set, the heat load can be imposed selecting the exposure time. A typical heat impulse is showed in Figure 1b.

In our test, an acetylene on oxidizer ratio equal to 1.33 was chosen in order to produce a neutral flame: this condition minimizes the thermo-oxidative ablation of the tested material satisfying the ASTM E-285-80 [1]. The in-depth temperature profiles were acquired at 3 mm (i.e., about 1/3 L, referred as T1) and 6 mm (i.e., about 2/3 L, referred as T2) from the hot surface on the corresponding isothermal surfaces. Fully sheathed, ungrounded, K-type thermocouples were placed in two blind holes which were drilled parallel to the exposed surface of the samples. To ensure a consistent acquisition of the temperatures, the depth of the holes was kept constant at 5 mm, i.e., in correspondence of the centerline of the sample, exactly where the torch impinged. A dual-band pyrometer (DIAS Infrared Systems model PYROSPOT DSR 55N) able to operate up to 3000 °C was used to record the surface temperature of the samples tested with the OAT (Figure 1c). It is worth noting that at a sample-to-pyrometer distance equal to 800 mm (as in our tests), the temperature on the exposed surface of the sample is read on an area (circle) having a diameter of about 3 mm i.e., in line with the size of the torch spot (see Figure 1a). 

Finally, the loss of mass due to the ablation process was also evaluated.

**Figure 2:**
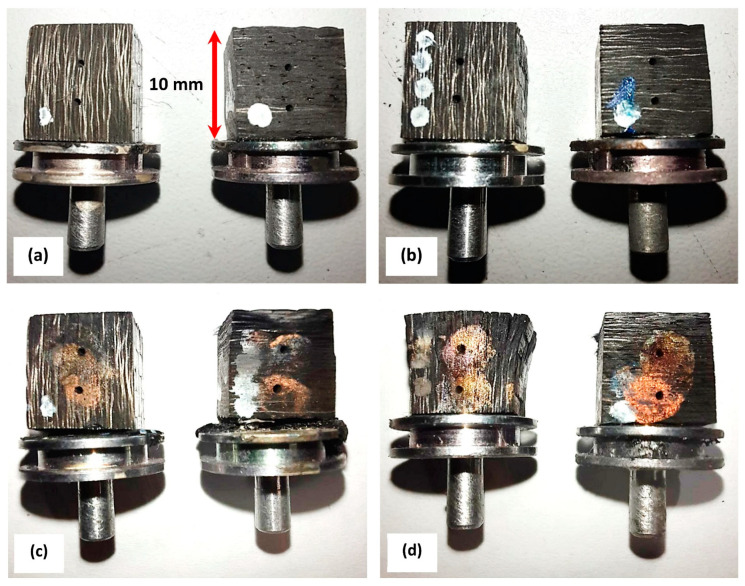
Virgin in-plane and out-plane OAT samples of the C-P (**a**) and C-P10E (**b**). Post-OAT testing appearance of the in-plane and out-plane samples of the C-P (**c**) and C-P10E (**d**).

Figure 2 reports the appearance of the C-P (a) and C-P10E (b) OAT virgin samples and the testing coupons of the same formulations after the torch test (C-P (c) and C-P10E (d)). For the out-plane tests, an exposure time of 30 s was selected. On the other hand, for the in-plane tests, an exposure time equal to 10 s was chosen due to the fact that the in-depth charring was quite faster than the out-plane case, as a result of the high thermal diffusivity of CPC samples oriented along the fiber direction. 

**Figure 3:**
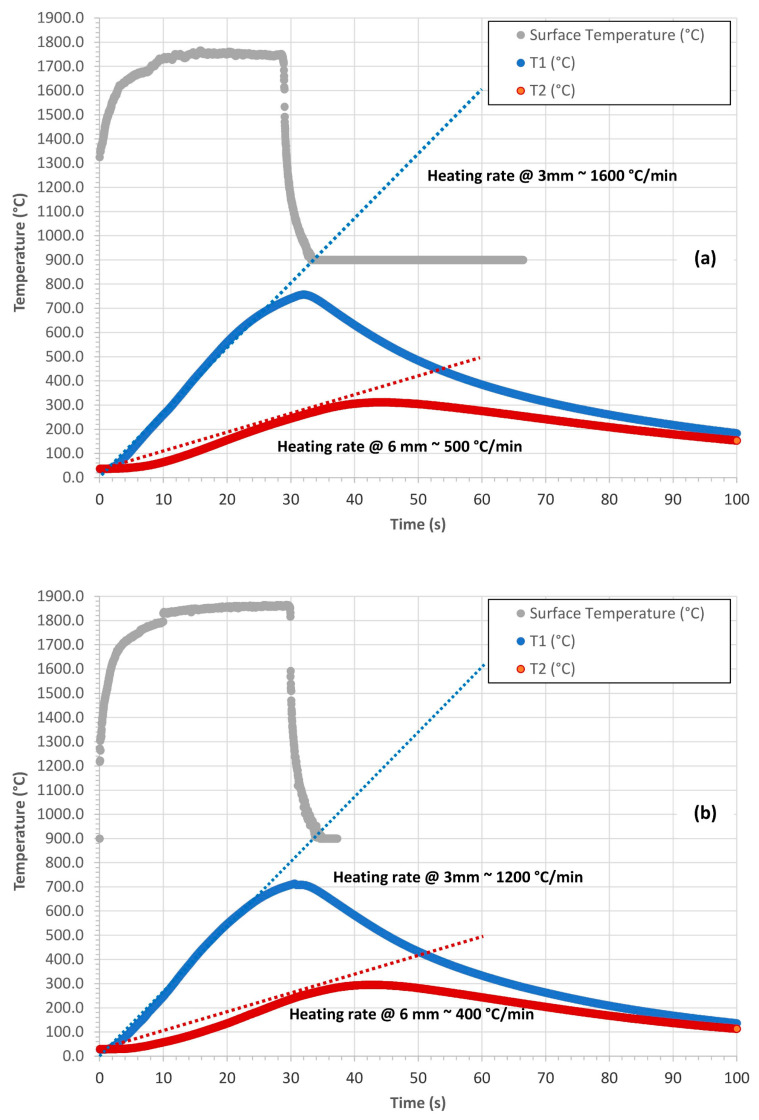
Out-plane temperature profiles of the C-P (**a**) and C-P10E (**b**). The approximate heating rates of the rising parts of the curves are also reported.

**Figure 4:**
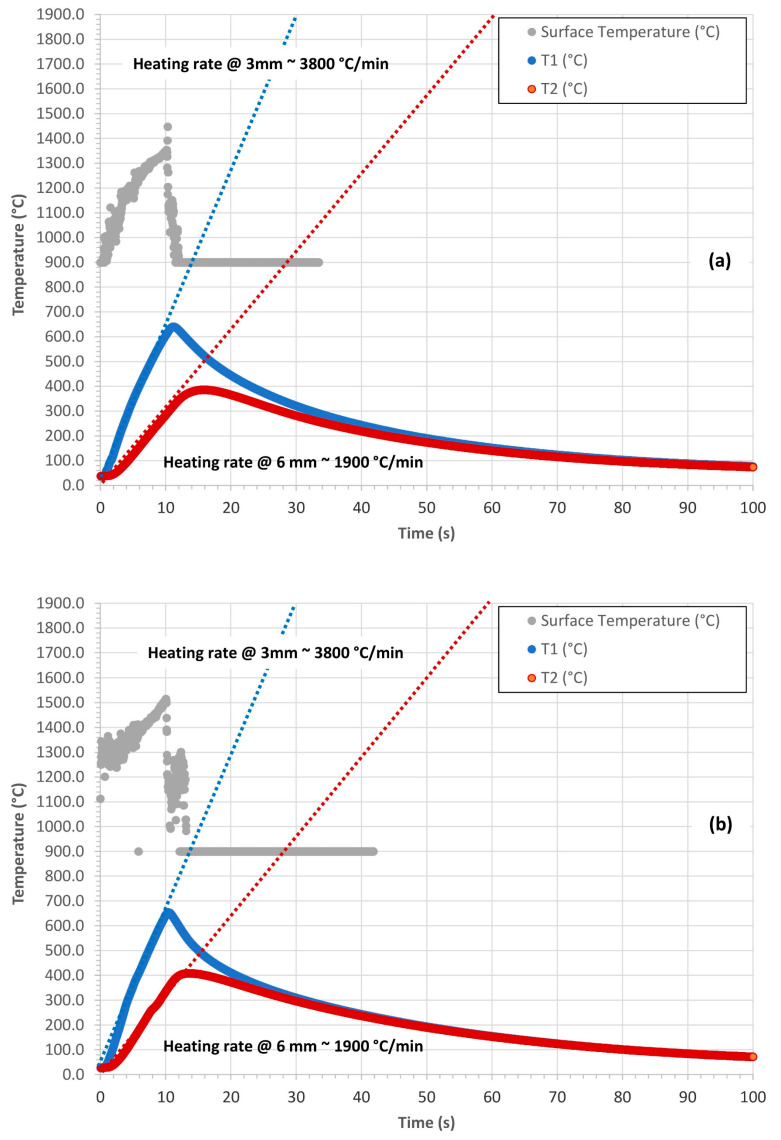
In-plane temperature profiles of the C-P (**a**) and C-P10E (**b**). The approximate heating rates of the rising parts of the curves are also reported.

The most representative temperature profiles of the surface temperature $T_{S}(t)$ , of the *T*1(*t*) (acquired at 3 mm from the surface) and of the *T*2(*t*) (acquired at 6 mm from the surface) of the C-P and C-P10E both in the out plane and in plane configurations are reported in Figure 3 and Figure 4.

From a qualitatively point of view, for both the C-P as well as for the C-P10E, considering the samples tested out plane, the approximate heating rate of the rising part of the temperature profile acquired at 3 mm—*T*1(*t*)—was of about 1600 °C/min (Figure 3a,b) while the approximate heating rate of the rising part of the temperature profile acquired at 6 mm—*T*2(*t*)—was of about 500 °C/min (Figure 3a,b). On the other hand, from a qualitatively point of view, for both the C-P as well as for the C-P10E, considering the samples tested in-plane, the approximate heating rate of the rising part of the temperature profile acquired at 3 mm—*T*1(*t*)—was of about 3800 °C/min (Figure 4a,b), while the approximate heating rate of the rising part of the temperature profile acquired at 6 mm—*T*2(*t*)—was of about 1900 °C/min (Figure 4a,b). The grey curves reported in Figure 3 and Figure 4 correspond to the surface temperature profiles registered by the pyrometer.

### 2.3. More Insights on the Heat Capacity of CPCs

**Figure 5:**
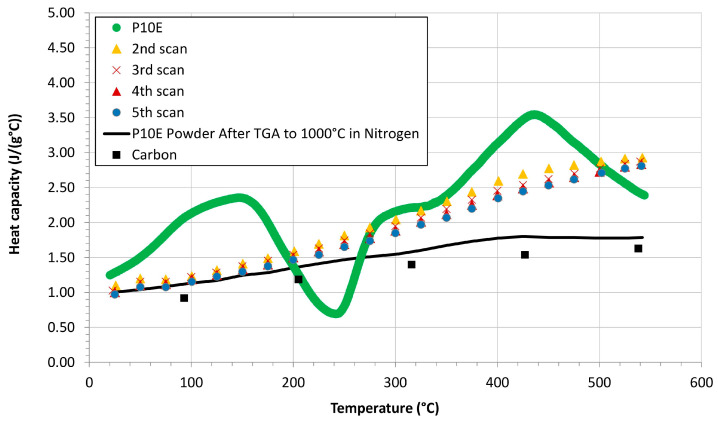
Heat capacity of P10E after four dynamic scans compared to the $C_{p}$ of carbon (Black ■ from [58]) and of the material obtained after a sixth scan in TGA test up to 1000 °C (black continuous line).

In general, the heat capacity of a CPCs can be considered composed by a reversible component (Debye or Einstein like [8]) with a superimposed irreversible part (degradation, i.e., charring of the polymeric matrix). To note the differences between the reversible and irreversible part of the heat capacity, and in order to qualitatively evaluate the degree of conversion of the matrix from an organic material to a carbonaceous medium, a cycle of four DSC tests (through a TA Instruments, model Q200 [31]) was carried out on the P10E matrix used to produce the C-P10E. For the first scan, the mass of the sample was considered temperature-dependent in Equation (2) (and obtained through a TGA test) while for all the following four scans, the mass of the material was considered constant and with a value equal to the original mass of the sample reduced by a factor given by the value of the TGA of the P10E matrix tested in nitrogen at 550 °C (all the technical details on this analysis are reported in [31]). Figure 5 shows that after a first scan, the original shape of the P10E is completely altered and the irreversible part of the heat capacity is basically no longer present. However, even after four additional scans operated at the maximum temperature allowed by our DSC (550 °C), the capability to convert the powdered P10E into a charred medium was limited. This conclusion can be drawn considering the values of the heat capacity of a fully carbon material [58] reported in the same figure (black dots) which were very different from the 5th scan on the P10E (blue curve). Since the maximum temperature allowed by the DSC used in this analysis was not sufficient to operate a change of the P10E in a fully charred medium, i.e., the organic-to-char conversion rate was below 1 [8], we carried out a TGA dynamic scan in nitrogen up to 1000 °C on the powders obtained at the end of the 5th scan and the resulting powders were studied again into the DSC. As reported in Figure 5, the TGA scan reduced the difference between the heat capacity of a fully carbon-based material (black dots) [58] and the charred powders of the P10E (black line), i.e., after the TGA test, the resulting degree of conversion was closer to 1. Consequently, at the end of all these tests, the original organic matrix is fully charred and the irreversible part of the $C_{p}$ has been completely removed.

**Figure 6:**
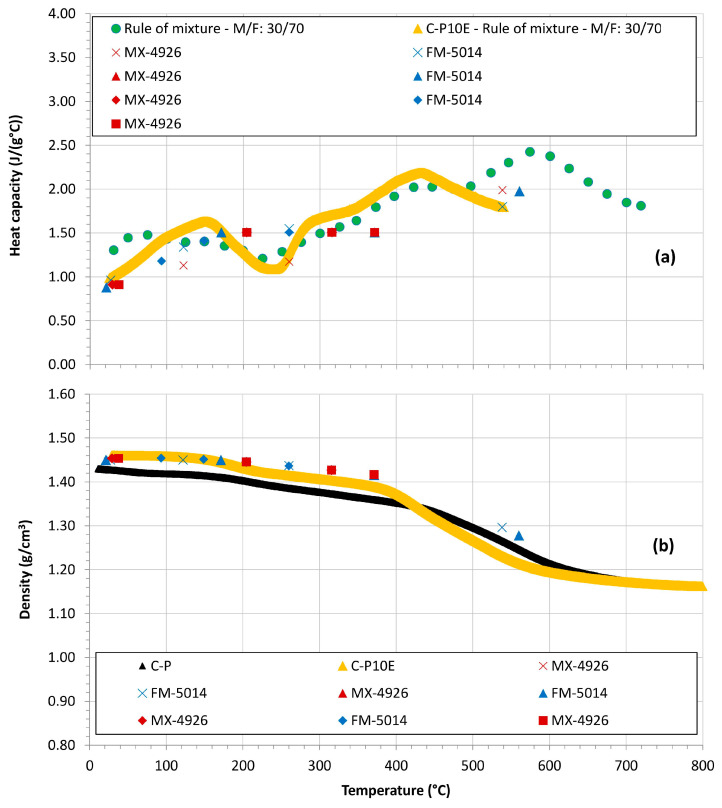
Heat capacity (**a**) and density (**b**) as a function of temperature for the developed CPCs as compared to state of art formulations. (Green ● from [32]; Yellow ▲ from [31]; Red and Blue **x** from [60]; Red and Blue ▲ from [61]; Red and Blue ⯁ from [62]; Red ■ from [63]).

Figure 6a reports the $C_{p}$ of the C-P10E as a function of temperature as compared to state-of-the-art CPC formulations. We also exploited the data of the heat capacity of a phenolic matrix studied by NASA in [32] and, through the rule of mixture [31], the $C_{p}$ of a CPC made of 30 wt% of carbon fiber and 70 wt% of matrix (this method was validated in [31,59]), was extrapolated. Figure 6b also reports the ρ of the C-P10E as a function of temperature as compared to state-of-the-art CPC formulations. In this figure, we considered an upper temperature limit of 800 °C, i.e., a value in which all the charring processes can be considered completed. 

**Figure 7:**
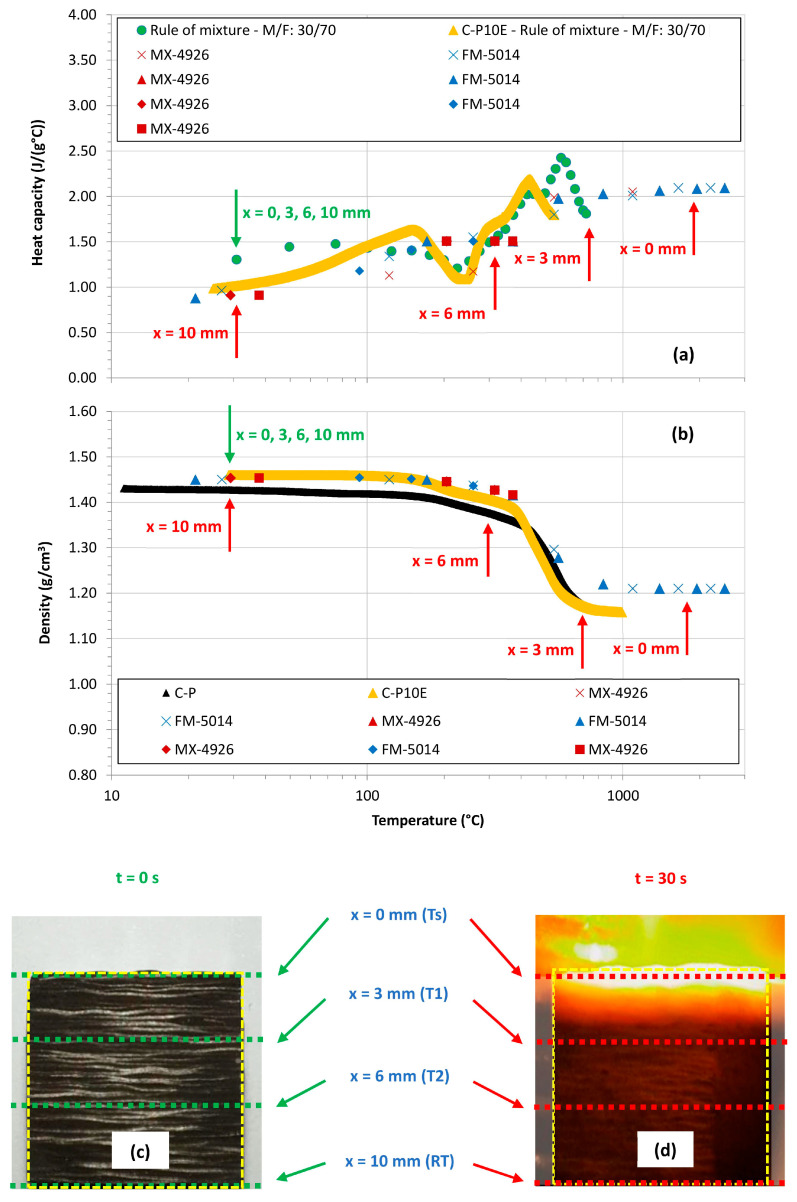
Heat capacity (**a**) and density (**b**) as a function of temperature for the developed CPCs as compared to state-of-the-art formulations up to 3000 °C. Sample of a CPC in the virgin state (**c**) and at the end of the heat impulse imposed by the OAT test (**d**). The different depths at which the temperatures are measured are reported and then correlated with the heat capacity and density profiles. (Green ● from [32]; Yellow ▲ from [31]; Red and Blue **x** from [60]; Red and Blue ▲ from [61]; Red and Blue ⯁ from [62]; Red ■ from [63]).

Moreover, Figure 7 reports the behavior of the $C_{p}$ and of the ρ of the materials considered in Figure 6 in the entire range of temperature typically considered to properly model the ablation response of CPCs, i.e., up to 3000 °C (in this case we were forced to use a log scale in the temperature axis). From these graphs, it is possible to evidence that in terms of heat capacity above about 800 °C, the value of the $C_{p}$ is considered to possess a gradually increasing, monotonic behavior with no presence of irreversible phenomena [8,9]. For the density of CPCs, it is possible to conclude that above about 800 °C, the value of the ρ of the CPC formulations tend to be considered constant as reported in the literature [60,61,62,63]. In general, in a cubic CPC sample exposed to the torch (Figure 7c), the thermal degradation depends on the depth of the sample we consider with the surface exposed to the harshest conditions (see in-depth temperature profiles in Figure 3 or Figure 4). As a result, assuming our OAT testing conditions, the degradation is highest on the surface of the sample while in the cold part of the test coupon, the material is still virgin (Figure 7c,d) and at room temperature (RT), i.e., the thermo-physical properties are not uniform through the sample. More in detail, if we consider the OAT testing condition reported in Section 2.2 for an out-plane CPC sample, it is possible to correlate the maximum temperatures recorded at 0 mm (*Ts*), 3 mm (*T*1), 6 mm (*T*2), and 10 mm (*RT*), at the beginning (0 s, Figure 7c) and at the end of the test (30 s, Figure 7d) with the values of the $C_{p}$ and of the ρ at these given temperatures (Figure 7a,b) evaluating the values of these properties at the given depths (Figure 7c,d). The green arrows are used to graphically indicate the values of the $C_{p}$ and of the ρ (in Figure 7a,b, respectively) at the beginning of the tests (Figure 7c), while the red arrows are used to graphically indicate the heat capacity and density of the same testing coupon (in Figure 7a,b, respectively) at the end of the OAT test (Figure 7d). At 0 s, independently from the depth, the values of the $C_{p}$ and of the ρ are constant ( $C_{p}(RT)$ and $\rho \left(RT\right),\;\forall$ depth), while at the end of the test (30 s), the values of these thermo-physical properties possess a dependence on the depth ( $C_{p}({Ts}_{max})$ and $\rho \left({Ts}_{max}\right)$ at 0 mm, $C_{p}({T1}_{max})$ and $\rho \left({T1}_{max}\right)$ at 3 mm, $C_{p}({T2}_{max})$ and $\rho \left({T2}_{max}\right)$ at 6 mm, and $C_{p}(RT)$ and $\rho \left(RT\right)$ at 10 mm).

**Figure 8:**
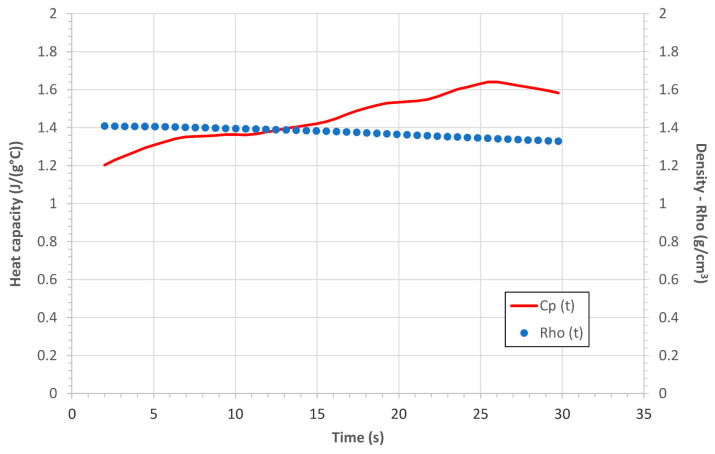
Mean values of heat capacity and density as a function of time of a C-P10E coupon tested with the OAT.

Since the values of the $C_{p}$ and of the ρ are not uniform through a CPC sample subjected to an OAT as explained in Section 2.2., if we want to evaluate the mean values of these thermo-physical properties of the entire tested coupon as a function of the exposure time (from 0 s to 30 s), once we define a mesh of the sample, we have to consider the contribution of each slice in terms of value of heat capacity and of density as a function of the temperature (or of the charring degree). As a result, if as an example we consider an out-plane sample of C-P10E exposed to the OAT for 30 s, if we evaluate the evolution of the mean $\langle C_{p}\rangle$ and $\langle \rho \rangle$ as function of the exposure time, then is possible to obtain the graphs reported in Figure 8.

Figure 8 demonstrates that even considering the dependence of $C_{p}$ and ρ with the temperature, the denominator of Equation (1) can be considered nearly constant, i.e., during the OAT test, the thermal diffusivity depends on the temperature manly through the k .

### 2.4. Theoretical Background on the Adopted Heat Conduction Models

#### 2.4.1. Ideal Semi-Infinite Solid

The proposed method to study the thermal diffusivity of CPCs is based on the assumption of the semi-infinite solid. Since in principle it extends infinitely in all directions except one, this solid is defined by a singular identifiable surface. If a sudden change in the conditions is imposed at this surface, transient, one-dimensional conduction will occur within the solid. The semi-infinite solid provides a useful idealization for many practical problems. Among the uniqueness of the protocol proposed in this paper it is worth mentioning the relative simplicity to run the experiments while allowing to carry out test that cannot be reproduced by any other common protocol designed to evaluate the thermal diffusivity such as an LFA (especially in terms of heating rates). 

**Figure 9:**
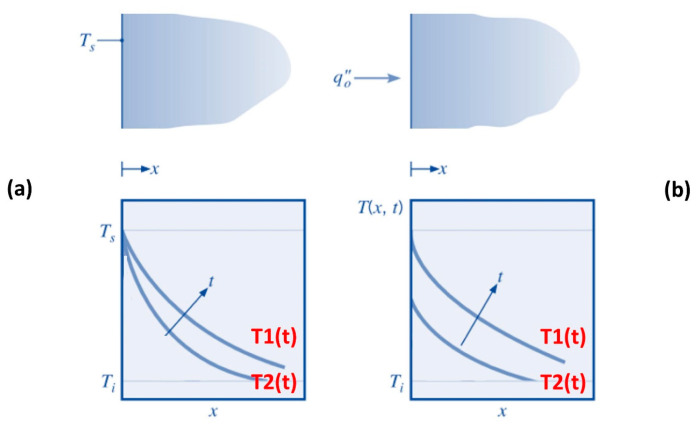
Transient temperature distributions in a semi-infinite solid for constant surface temperature (**a**) and constant heat flux (**b**).

Two scenarios will be considered in this approach: constant surface temperature or constant heat flux [64]. The first scenario of constant surface temperature is summarized in Figure 9a and responds to the following equation:

(3) $$\frac{T\left(x,t\right)-T_{S}}{T_{i}-T_{S}}=erf\left(\frac{x}{2\sqrt{\alpha t}}\right)$$

where erf is the error function, $T_{i}$ is the temperature of the slab at t=0 , $T_{S}$ is the surface temperature imposed by the torch plume, $T\left(x,t\right)$ is the temperature recorded at x=3 mm or *T*1(*t*) and at x=6 mm or *T*2(*t*) and α is the thermal diffusivity of the tested expressed as in Equation (1). In most of the cases, Equation (3) is used to derive the in-depth temperature profiles $T\left(x,t\right)$ of a given material exposed to a constant surface temperature once the thermal diffusivity of the given material is known:

(4) $$T\left(x,t\right)=erf\left(\frac{x}{2\sqrt{\alpha t}}\right)\left(T_{i}-T_{S}\right)+T_{S}.$$

The surface net heat flux $q_{net}^{\prime \prime }$ can be obtained by applying Fourier’s law at x=0 , in which case:

(5) $$q_{net}^{\prime \prime }=-k{\left.\frac{\partial T}{\partial x}\right|}_{x=0}⟶q_{net}^{\prime \prime }=\frac{k\left(T_{S}-T_{i}\right)}{{\left(\alpha t\right)}^{\frac{1}{2}}}{\left(\frac{1}{\pi }\right)}^{\frac{1}{2}}=\frac{\alpha \rho C_{p}\left(T_{s}-T_{i}\right)}{{\left(\alpha t\right)}^{1/2}}{\left(\frac{1}{\pi }\right)}^{\frac{1}{2}}$$

where k is the mean thermal conductivity of the sample. From the knowledge of the $T_{S}(t)$ (acquired with a pyrometer as described in Section 2.2), of the *T*1(*t*) and of the *T*2(*t*) (acquired with thermocouples as described in Section 2.2) and exploiting only the heating phase of the test (Figure 3 and Figure 4), Equation (3) can be solved to retrieve α . 

The second scenario of constant heat flux is summarized in Figure 9b and responds to the following equation:

(6) $$T\left(x,t\right)-T_{i}=\frac{2q_{0}^{\prime \prime }\sqrt{\frac{\alpha t}{\pi }}}{k}\exp\left(\frac{{-x}^{2}}{4\alpha t}\right)-\frac{q_{0}^{\prime \prime }x}{k}erfc\left(\frac{x}{2\sqrt{\alpha t}}\right)$$

where $T_{i}$ is the temperature of the slab at t=0. It is worth remembering that the complementary error function, erfcw , is defined as erfcw≡1−erfw , and $q_{0}^{\prime \prime }$ is the surface heat flux which can be obtained by applying Fourier’s law at *x* = 0, in which case:

$$q_{net}^{\prime \prime }=q_{0}^{\prime \prime }.$$

As for the first scenario, from the knowledge of the experimental values of the $T_{S}(t)$ (acquired with a pyrometer as described in Section 2.2), of the *T*1(*t*) and of the *T*2(*t*) (acquired with thermocouples as described in Section 2.2) and exploiting only the heating phase of the test (Figure 1), Equation (6) can be numerically solved to evaluate the α (T) . In Equation (6), the thermal conductivity can be expressed as $k=\alpha \;\rho {\;C}_{p}$ and, as a result, from the knowledge of the $T\left(x,t\right)$ , this equation can be solved in α as a function of the temperature directly from the knowledge of ρ and of the $C_{p}$ :

(7) Tx,t=q0′′k2αtπ exp⁡−x24αt−xerfcx2αt+Ti.

Considering that $k=\alpha \;\rho {\;C}_{p}$ , Equation (7) can be directly expressed in terms of $\alpha \;\rho {\;C}_{p}$ :

Tx,t=q0′′αρCp2αtπ exp⁡−x24αt−xerfcx2αt+Ti.

Even though Equation (7) has been found considering a constant surface heat flux, we wanted to relate $q_{net}^{\prime \prime }$ on the $T_{S}\left(t\right)$ . An explicit expression for the $q_{net}^{\prime \prime }$ as a function of the $T_{S}\left(t\right)$ can be obtained from Equation (6) evaluating the $T\left(x,t\right)$ on the surface:

(8) $$T\left(0,t\right)-T_{i}=\frac{q_{net}^{\prime \prime }}{k}\left(2\sqrt{\frac{\alpha t}{\pi }}\right)$$

but since $T\left(0,t\right)$ is the $T_{S}\left(t\right)$ , Equation (8) can be rewritten as:

$$k\left(T_{S}\left(t\right)-T_{i}\right)=q_{net}^{\prime \prime }{\left(\frac{4\alpha t}{\pi }\right)}^{\frac{1}{2}}.$$

Then, the $q_{net}^{\prime \prime }$ can be expressed in terms of $T_{S}\left(t\right)$ as follows:

(9) $$q_{net}^{\prime \prime }=\frac{k\left(T_{S}\left(t\right)-T_{i}\right)}{{\left(\alpha t\right)}^{\frac{1}{2}}}{\left(\frac{\pi }{4}\right)}^{\frac{1}{2}}.$$

As a result, if we compare Equations (5) to (9), we can see that in the expression of the ${\;q}_{net}^{\prime \prime }$ there is a common term:

$$\frac{k\left(T_{S}\left(t\right)-T_{i}\right)}{{\left(\alpha t\right)}^{\frac{1}{2}}}$$

times the term ${(1/\pi )}^{1/2}$ in the constant surface temperature scenario, or times ${(\pi /4)}^{1/2}$ in the case of the constant heat flux framework. 

Ideally, in a material in which the thermo-physical properties are constant, with a step change in the surface temperature (case 1), the temperatures within the medium monotonically approach $T_{s}$ with increasing t, while the magnitude of the surface temperature gradient, and hence the surface heat flux, decreases as $t^{-1/2}$ . On the other hand, for a constant surface heat flux (case 2), Equation (6) reveals that $T\left(0,t\right)=T_{s}\left(t\right)$ increases monotonically as $t^{1/2}$ .

#### 2.4.2. Heat Transfer Models in Presence of Non-Constant Thermo-Physical Properties

In a polymeric ablative material such a CPC the dependence of α , ρ and of the ${\;C}_{p}$ from the temperature (they are not constant) alter the shape of the temperature profiles. As an example, for a CPC, the extent of the irreversible part of the heat capacity profile of a phenolic matrix which is associated with the decomposition (or charring) of the matrix and which works as a heat sink, influences the in-depth temperature profile of the composite virgin/charred material. Equation (4) is analytically derived from the assumption of constant surface temperature and constant thermo-physical properties. Moreover, the above introduced models also imply uniform α through the thickness of the sample. Nonetheless, it has been showed that this analytical model can be used also in the case of non-constant α , i.e., $\alpha (T)=k(T)/\rho \left(T\right)C_{p}\left(T\right)$ [65]. We will show that with the proper assumptions, the same conclusion can be drawn for Equation (7).

It should be pointed out that the value of α determined from this type of calculation is not the classical thermal diffusivity that is a conduction property of a material as summarized in Equation (1). Rather, it is a pseudothermal diffusivity that includes the effects of conduction, chemical reactions within the char, and, most important, radiation within the char. The value of α determined from Equations (4) or (7) starting from the in-depth temperature profile is the average value between the exposed surface ( $T_{S}$ ) and the internal isotherm ( $T\left(x,t\right)$ ) for which the thermal diffusivity is being calculated [65]. As a result, from a mathematical point of view, keeping in mind the considerations introduced in Section 2.2, the values of the thermal diffusivity derived from Equations (4) or (7) can be considered as mean values ( $\langle \alpha \rangle$ ) of the region comprised between the surface and the given ( x ). However, in terms of heat transfer analysis the mean values of the thermal diffusivity determined from Equations (4) or (7) keep into account the effect of the entire sample.

## 3. Results and Discussion

### 3.1. Post-OAT Appearance of the Tested CPCs

**Figure 10:**
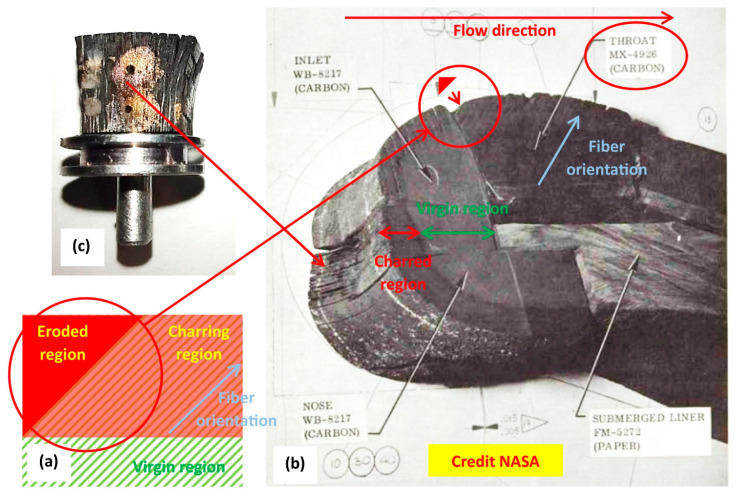
Charring phenomena within the CPC walls of a nozzle of a solid rocket motor (**a**). Post-burning appearance of a composite segmented submerged nozzle assembly made of CPCs and related phenomena of erosion and delamination (**b**). Appearance of an in-plane CPC sample tested with the OAT reproducing the delamination processes evidenced in (**c**), i.e., in the upper portion of the throat made of MX-4926 (image produced starting from an image taken from [66], credit NASA).

Even though this paper is focused on the evaluation of the thermal diffusivity of CPCs, some comments on the post-OAT appearance of the tested formulations will be made. In fact, in a previous paper [2], we assessed the dependence of the Inter Laminar Shear Strength (ILSS) of the C-P and C-P10E formulations as a function of the temperature. In [2], it was reported the positive effect of the epoxy phase in the phenolic matrix in terms of capability to boost the ILSS of the resulting composites. However, in that study, we did not focus the attention on the capability of the C-P and C-P10E formulations tested in-plane with the OAT. According to Figure 2, it is possible to evidence that in the in-plane OAT tests the neat C-P tended to behave better than the C-P10E since the sample of the hybrid epoxy–phenolic-based CPC tended to be more delaminated in correspondence of the exposed surface. This behavior which can be evidently attributed to the polymeric phase, i.e., once fully charred, the hybrid epoxy–phenolic matrix P10E tended to degrade more than the neat phenolic matrix and, in other terms, even the addition of 10 wt% of epoxy on the phenolic matrix tended to reduce the char yield of the resulting P10E. This effect could not be directly evidenced in the out-plane with hybrid virgin/charred coupons studied in the out-plane configuration in [2,3]. To better understand these results, it is worth noting that during the firing of the motor [66] the inner layers of the nozzle wall start to char (Figure 10a, in red) dramatically reducing the local ILSS (over 90%). As a result, the structural integrity of the nozzle wall is enabled by the virgin portion (Figure 10a, in green) of the overlapping fiber layers. The charred part does not contribute to the structural integrity of the nozzle wall which is subjected to ply-lift. Depending on the combustion time, every design and layout of the fibers have to keep into account the fact that a certain portion of the CPC wall has to be left virgin to avoid the failure of the nozzle. That is the reason why it is imperative to have CPCs with the highest ILSS and the lower thermal conductivity. The part of throat evidenced in the circular red area of Figure 10b is completely charred as compared to other portions of the same part which still possess a certain level of virgin material—as resumed in Figure 10a. In Figure 10b, the triangular part evidenced in red is the portion that does not possess any virgin material and thus is completely removed. 

Consequently, although in the in-plane OAT tests, the fully charred formulation C-P10E appears worse than the completely carbonized C-P neat material (in terms of delamination), it is worth remarking that the erosion resistance of the given part of the SRM nozzle assembly has to be evaluated in the hybrid virgin/charred configuration (as in Figure 10a). In other words, the overall delamination (especially in the in-plane configuration as reported in Figure 10c) and erosion resistance of the given item depend on the ratio between the ILSS of the charred CPC as compared to the ILSS of virgin backup material (Figure 10b). With this respect, since this paper is not focused on the study of the relative merit of the C-P as compared to the C-P10E in terms of ILSS, a discussion on the loss of the mechanical properties of fully charred samples of CPCs (under isothermal condition, i.e., similarly to the in-plane coupons studied in this research) as compared to hybrid virgin/charred coupons (out-plane) has been addressed in [2].

### 3.2. Evaluation of the Thermal Diffusivity: Constant Surface Temperature Scenario

**Figure 11:**
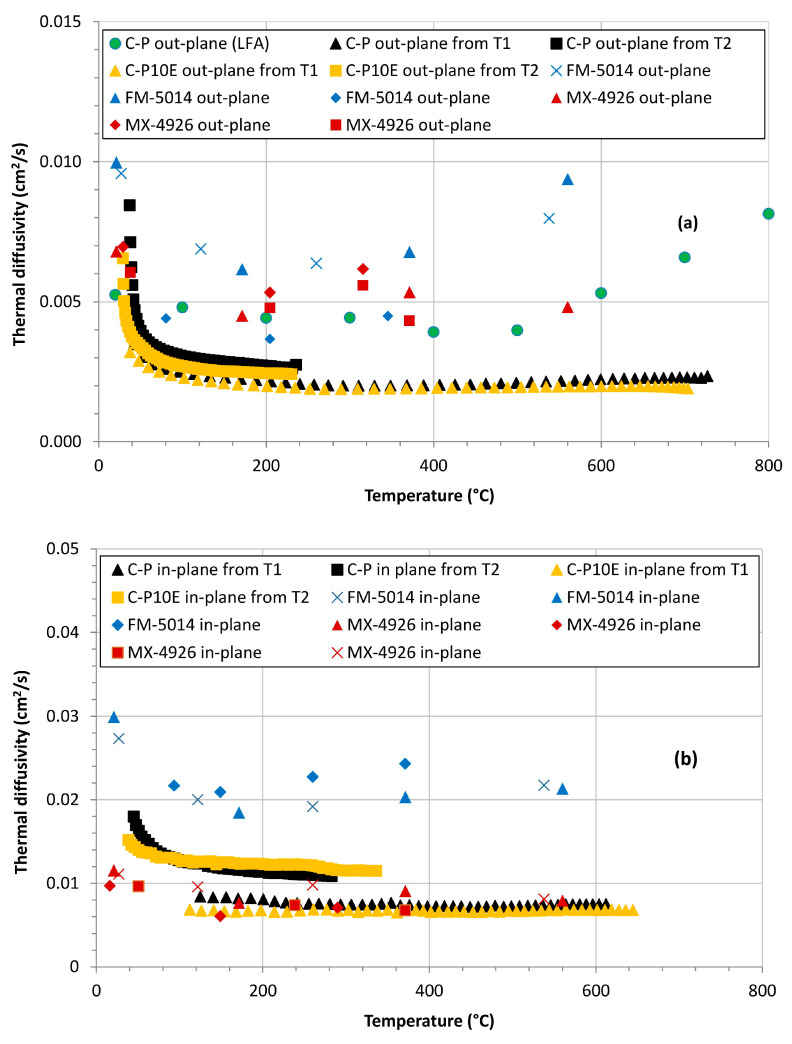
Out-plane thermal diffusivity of the C-P and C-P10E formulations obtained under the assumption of constant surface temperature as compared to the corresponding data of the state-of-the-art counterparts (MX-4926 and FM-5014) (**a**). In-plane thermal diffusivity of the C-P and C-P10E formulations obtained under the assumption of constant surface temperature as compared to the corresponding data of the state-of-the-art counterparts (MX-4926 and FM-5014) (**b**). (Red and Blue **x** from [60]; Red and Blue ▲ from [61]; Red and Blue ⯁ from [62]; Red ■ from [63]).

The most representative temperature profiles of each formulation C-P and C-P10E, in the in-plane and out-plane configurations (Figure 3 and Figure 4) were used to evaluate the in-plane and out-plane thermal diffusivity through Equation (3). The numerical solution of this equation with MATLAB (R2023a) did not imply any particular difficulty. Figure 11 reports the out-plane thermal diffusivity α (T) obtained with these OAT test exploiting the *T*1(*t*) and *T*2(*t*) temperature profiles, as compared to the corresponding data referred to the MX-4926 and FM-5014 [60,62,63,67,68]. The out-plane thermal diffusivity of the C-P formulation evaluated with an LFA test is also reported (unfortunately, the LFA results in-plane are not available). In the range of temperatures considered in our LFA tests, the C-P and the C-P10E have an out-plane thermal diffusivity qualitatively in line with the state-of-the-art CPC formulations. However, from a quantitative point of view, when adopted to retrieve the thermal diffusivity, Equation (3) produced numerical results lower than the values of state-of-the-art CPCs (such as the FM-5014) manufactured with low-fired stretch-broken PAN-CFs and even of those made with Rayon-CFs (such as the MX-4926). Moreover, the obtained curves of α (T) tended to exhibit a dependence from the temperature less marked than the in the case of the MX-4926 and FM-5014, also considering that, in principle, the CPCs tested in this research should exhibit a thermal diffusivity higher than the values referred to the FM-5014. These results encouraged further efforts to increase the effectiveness of the protocol aimed at retrieving the thermal diffusivity using the in-depth temperature profiles and using the modified constant heat flux scenario.

### 3.3. Evaluation of the Thermal Diffusivity: Modified Constant Heat Flux Scenario

**Figure 12:**
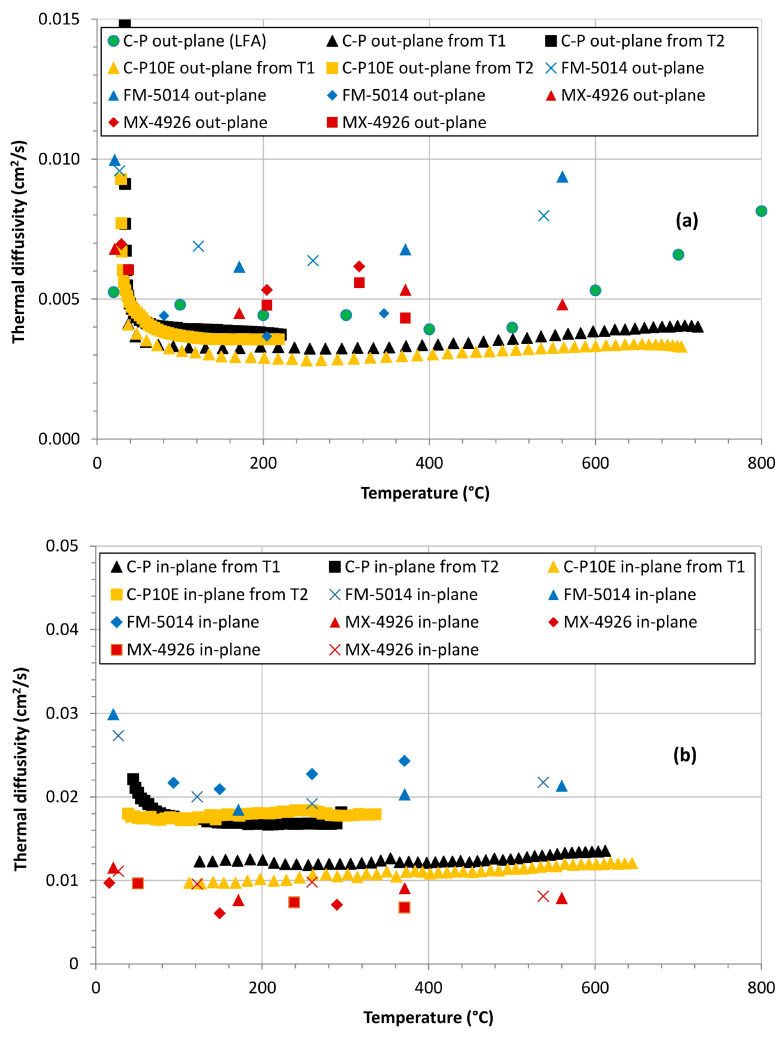
Out-plane thermal diffusivity of the C-P and C-P10E formulations obtained under the assumption of constant surface heat flux as compared to the corresponding data of the state-of-the-art counterparts (MX-4926 and FM-5014) (**a**). In-plane thermal diffusivity of the C-P and C-P10E formulations obtained under the assumption of constant surface heat flux as compared to the corresponding data of the state-of-the-art counterparts (MX-4926 and FM-5014) (**b**). (Red and Blue **x** from [60]; Red and Blue ▲ from [61]; Red and Blue ⯁ from [62]; Red ■ from [63]).

As for the constant surface temperature scenario, the most representative temperature profiles of each formulation C-P and C-P10E, in plane and out-plane (Figure 3 and Figure 4) were used again to evaluate the out-plane and in-plane thermal diffusivity through Equation (7). The numerical solution of this equation with MATLAB resulted in being more difficult than the previous case. In fact, analytically speaking, while for the determination of α , Equation (3) basically depends on the experimental values of x, $T_{S}(t)$ , $T_{1}(t)$ and $T_{2}\left(t\right)$ , on the other hand, Equation (7) explicitly also keeps into account the density ρ(T) , the heat capacity $C_{p}(T)$ , as well as the values of the net surface heat flux $q_{net}^{\prime \prime }$ on the surface of the sample, increasing the difficulties to determine the values of α . However, as reported in Figure 8, since the term $\rho {(T)C}_{p}(T)$ tends to be nearly constant during the entire exposure time of the OAT test, most of the criticalities on the determination of α though Equation (7) is related to the values of the net surface heat flux $q_{net}^{\prime \prime }$ . Due to the difficulties in solving Equation (7) using the correct values of $q_{net}^{\prime \prime }$ and the reading of the heat flux from the slug calorimeter, we addressed the possibility of using Equation (9) for the determination of the net surface heat flux. In other words, since we did not explicitly know the exact values of the $q_{net}^{\prime \prime }$ , we decided to correlate the values of the net surface heat flux to the surface temperature $T_{S}(t)$ through Equation (9) also in the constant heat flux scenario. As a result, on the surface of the exposed sample, if we introduce Equation (9) into Equation (7), we analytically obtain Equation (10):

(10) $$T\left(x,t\right)=\left(T_{S}\left(t\right)-T_{i}\right)\left(\exp\left(\frac{{-x}^{2}}{4\alpha t}\right)-{\left(\frac{\pi }{4\alpha t}\right)}^{\frac{1}{2}}xerfc\left(\frac{x}{2\sqrt{\alpha t}}\right)\right)+T_{i}$$

i.e., a new equation which correlates the $q_{net}^{\prime \prime }$ at the $T_{S}(t)$ as in the first scenario. Solving this equation, we obtain the results reported in Figure 12.

Figure 12 shows that the values of thermal diffusivity obtained in the second scenario are higher than the corresponding obtained in the constant surface temperature framework, i.e., they align more closely with the values derived from the LFA, highlighting the relatively superior predictive abilities of the modified constant heat flux model. However, especially for the out-plane samples, in both scenarios it is possible to see that above about 600 °C the results from our protocol tend to be lower than the values obtained from LFA or from the literature. This difference can be explained as follows.

## 4. Concluding Considerations

Starting from the equations for the in-depth temperatures obtained in the framework of semi-infinite solid and constant surface temperature and constant heat flux, two protocols aimed at retrieving the thermal diffusivity as a function of temperature of carbon/phenolic composites tested with an oxy-acetylene torch were validated. Both models showed to be feasible and exploitable to determine the thermal diffusivity of CPCs starting from the sole experimental in-depth temperature profiles. Up to a temperature of about 600 °C, the values of thermal diffusivity of the studied CPCs were found to be consistent with state-of-the-art formulations (MX-4926 and FM-5014) both in the in-plane as well as in the out-plane configurations.

The first reason why in general the experimental values of the thermal diffusivity tend to show a limited dependency from the temperature above about 600 °C is due to the fact that when Equations (4) and (10) are used to numerically retrieve α , these relationships are not able to directly find a function that strongly depends on the temperature. In other words, once Equations (4) or (10) is solved, i.e., reversed to determine α starting from the given temperature profiles, both models (constant surface temperature or constant heat flux) tend to produce (at the given time t ) a thermal diffusivity value that fits for the whole sample independently from the degradation degree. At the give time, and depth (temperature), these equations work determining a single value of thermal diffusivity that, on the other hand, is the same for the entire sample. To make it very short, Equations (4) and (10) derive from the assumption of constant thermal diffusivity. This point will be further addressed in another (second forthcoming) paper.

**Figure 13:**
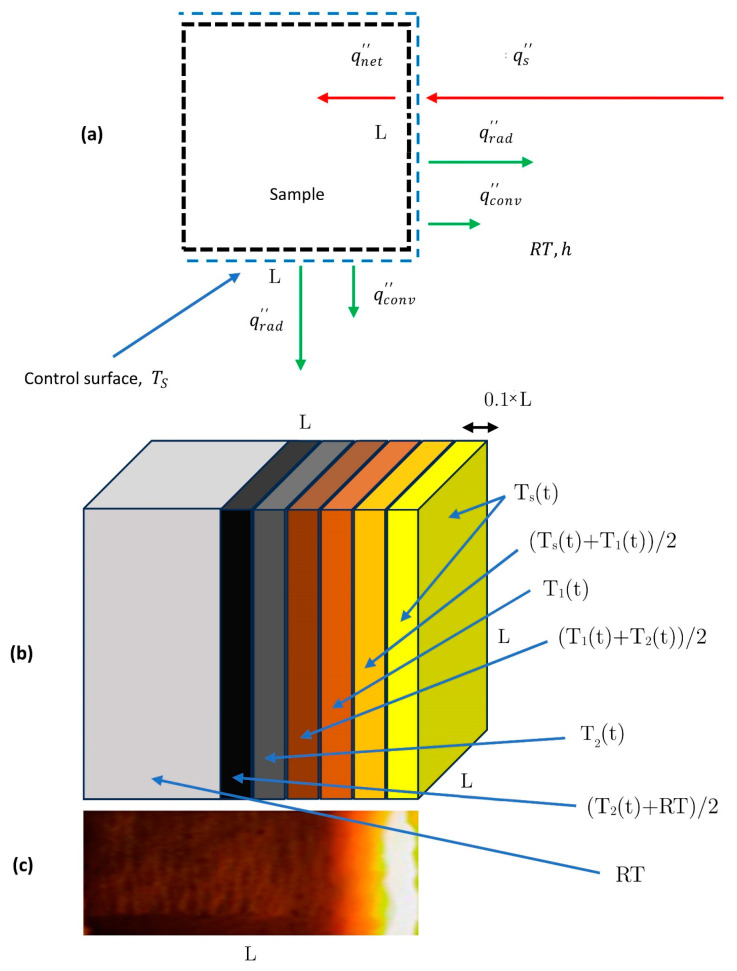
Control surface for the transient heat transfer problem (**a**). Possible schematization of the heat losses by radiation and convection on the surface and lateral faces of the square shaped sample (**b**). Real temperature distribution on the lateral surfaces of a tested cubic CPC sample (**c**).

The second main limitation related to our experiments is that considering samples like the ones reported in Figure 2—which are significantly different from being regarded as a semi-infinite solid—the experimental in-depth temperature profiles tend to be lower than in the ideal case. In fact, Figure 13a reports the boundary conditions in terms of input and output heat fluxes (or better power) for the exposed sample during the OAT test, i.e., when the torch is turned on [69]:

(11) $$q_{net}^{\prime \prime }A_{i}=q_{s}^{\prime \prime }A_{i}-q_{rad}^{\prime \prime }A_{o}-q_{conv}^{\prime \prime }A_{o}$$

where $q_{net}^{\prime \prime }$ is the net heat flux that enters in the CPC sample at the surface x=0 , $q_{s}^{\prime \prime }$ is the heat flux of the torch, and $q_{rad}^{\prime \prime }$ and $q_{conv}^{\prime \prime }$ are the heat fluxes losses by radiation and convection, $A_{i}$ is the surface in which we consider entering the heat flux ( $A_{i}=L^{2}$ , i.e., the surface of a face of the cubic sample) and $A_{o}$ is the surface that we consider responsible for the output of the heat flux since we are not in an ideal condition of semi-infinite solid.

Equation (11) defines the net heat flux $q_{net}^{\prime \prime }$ entering into the CPC sample tested with an OAT having an exposed surface at the temperature ${T\left(0,t\right)=T}_{s}(t)$ . $q_{net}^{\prime \prime }$ is then defined as a difference between $q_{s}^{\prime \prime }$ , i.e., the heat flux entering in the surface from the energy source (OAT) and the heat loss due to radiation ( $q_{rad}^{\prime \prime }$ ) and convection ( $q_{conv}^{\prime \prime }$ ). 

As an example, if we set the entire output surface $A_{o}$ as equal to $A_{i}+6\times 4\times 0.1\times A_{i}$ (see Figure 13b) and each slice of the cubic sample lateral surface is put at a temperature as reported in the same figure—trying to mimic the real in-depth (and lateral, supposing to have perfectly plane iso-thermal surfaces for the tested CPC sample (Figure 13c)—the contribution of the different heat fluxes in Equation (11) could be defined as follows:

(12) $$q_{net}^{\prime \prime }\left(t\right)A_{i}=q_{s}^{\prime \prime }\left(t\right)A_{i}+\;-\varepsilon \sigma \left(T_{s}^{4}\left(t\right)-T_{surr}^{4}\right)A_{i}+\;-\left[\left(\varepsilon \sigma \left({T_{s}\left(t\right)}^{4}-T_{surr}^{4}\right)A_{L}\right)+\left(\varepsilon \sigma \left({\left(\frac{T_{s}\left(t\right)+T1\left(t\right)}{2}\right)}^{4}-T_{surr}^{4}\right)A_{L}\right)\;+\left(\varepsilon \sigma \left({T1\left(t\right)}^{4}-T_{surr}^{4}\right)A_{L}\right)+\left(\varepsilon \sigma \left({\left(\frac{T1\left(t\right)+T2\left(t\right)}{2}\right)}^{4}-T_{surr}^{4}\right)A_{L}\right)\;+\left(\varepsilon \sigma \left({T2\left(t\right)}^{4}-T_{surr}^{4}\right)A_{L}\right)+\left(\varepsilon \sigma \left({\left(\frac{T2\left(t\right)+RT}{2}\right)}^{4}-T_{surr}^{4}\right)A_{L}\right)\right]+\;-h\left(T_{s}\left(t\right)-T_{surr}\right)A_{i}+\;-\left\{h\left(T_{s}\left(t\right)-T_{surr}\right)A_{L}+h\left(\frac{T_{s}\left(t\right)+T1\left(t\right)}{2}-T_{surr}\right)A_{L}+h\left(T1\left(t\right)-T_{surr}\right)A_{L}\;+h\left(\frac{T1\left(t\right)+T2\left(t\right)}{2}-T_{surr}\right)A_{L}+h\left(T2\left(t\right)-T_{surr}\right)A_{L}\;+h\left(\frac{T2\left(t\right)+RT}{2}-T_{surr}\right)A_{L}\right\}$$

where $A_{i}$ is the surface in which we consider entering the heat flux, while each isothermal lateral slice defined as $A_{L}=4*0.1*L*L=0.4*A_{i}$ —in which we consider exiting the heat flux—is set at a specific temperature as reported in Equation (12), ε is the emissivity of the tested material, σ is the Stefan–Boltzmann constant, h is the average surface convective heat transfer (which was determined experimentally from the cooling part of the temperature profiles), $T_{surr}$ , the surrounding temperature of the sample, corresponds to the room temperature ( $T_{surr}=RT$ ). In other words, in our example of possible loss of energy of the real sample (as compared to a semi-infinite solid), to properly assess the heat loss due to the lateral faces of the tested coupon, the contribution of Equation (12) between square brackets is the entire loss by radiation while the contribution between brace brackets is the entire loss by convection.

Starting from Equations (11) and (12), we preliminary determined that the sum of the heat loss (power loss) term between square brackets ( $q_{rad}^{\prime \prime }\;A_{L}$ ) and of the contribution between brace brackets ( $q_{conv}^{\prime \prime }\;A_{L}$ ) are extremely relevant and can also be substantially higher that the net entering heat term ( $q_{net}^{\prime \prime }A_{i}$ ). This evidence would support the considerations on the lower temperature profiles related to the real samples as compared to the ideal semi-infinite slab.

**Figure 14:**
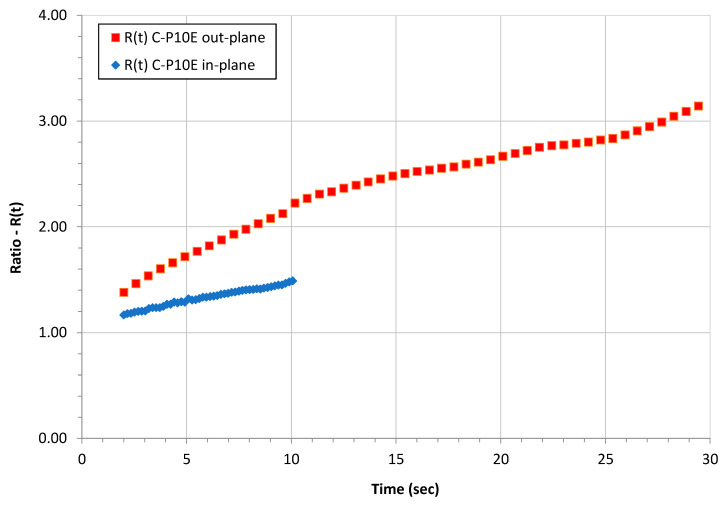
Behavior of the R(t) for the C-P10E out-plane and in-plane using the constant heat flux scenario.

In fact, starting from the assumptions at the beginning of this section on the heat losses summarized in Figure 13, if we define the term ratio as

$$R(t)=\frac{q_{net}^{\prime \prime }{(t)A}_{i}+{(q}_{rad}^{\prime \prime }(t)+q_{conv}^{\prime \prime }(t){)A}_{L}}{q_{net}^{\prime \prime }{(t)A}_{i}}$$

and we plot it as a function of the time for the C-P10E out-plane and in-plane using the constant heat flux scenario data, we obtained the graph presented in Figure 14. For the C-P10E out-plane, the loss of heat on the lateral surfaces of the sample is higher than in the case of the in-plane test. This result can be justified considering that the in-plane thermal diffusivity of the C-P10E is higher than the out-plane counterpart. In other words, as compared to the C-P10E in-plane samples, for the out-plane coupons a higher amount of heat is spread towards the lateral faces of the samples and then dissipated by radiation and convection on the four lateral faces of the coupon. This behavior would also imply the general better capability of the protocol to determine the thermal diffusivity in the case of the in-plane samples as compared to the out-plane coupons.

As a conclusion, it is worth highlighting that in the scenario of an ideal semi-infinite slab, all the temperatures introduced in this work ( $T_{s}\left(t\right)$ , $T1\left(t\right)$ , $T2\left(t\right)$ ) would be rescaled and increased thus modifying the values of thermal diffusivity. We would expect that a higher value of $T1\left(t\right)/T_{s}\left(t\right)$ would increase the value of α . In a forthcoming additional paper on the same topic, we will try to evaluate the effect of the loss of heat related to the lateral faces of the cubic sample on the values of the thermal diffusivity.

In further research, we will also try to consider the effect of the pyrolysis gases on the in-depth temperature profiles. In fact, phenolics possess a substantial amount of carbon-hydrogen bonds, which supply the evolution gases (such as H_2_ and CH_4_ and others) that contribute to the reduction in the local temperature and also provide the transpiration cooling [70,71,72,73,74,75,76,77]. At the same time, once the gas reached the surface, this gaseous film also works as a reaction barrier; however, when this gaseous layer is removed, due to the interaction of the hyper-thermal environment gases, chemical erosion tends to increase.

## 5. Conclusions

A protocol aimed at determining the thermal diffusivity of Carbon/Phenolic Composites (CPCs) through the Oxy-Acetylene Torch (OAT) tests was validated and presented in this paper. The proposed approach was demonstrated to bypass some limitations of common or commercial protocols, especially in terms of capability in determining the thermal diffusivity of CPCs at high heating rates and through experiments which reconstruct the real hyperthermal conditions of the TPS material. The proposed method to retrieve the thermal diffusivity of CPCs is based on the assumption of the semi-infinite solid. Two scenarios were considered in this approach: constant surface temperature or constant heat flux. The values of in-plane and out-plane thermal diffusivity measured for some experimental CPC laminates based on Polyacrylonitrile (PAN) based on Carbon Fibers (PAN-CFs) fabric resulted to be in line with the values of a state-of-the-art CPC such as the FM-5014 (manufactured with low-fired stretch-broken PAN-CFs). Finally, a thorough comparison of the comparison of the obtained data with state-of-the-art CPCs such as MX-4926 and FM-5014 has also been carried out.

## Figures and Tables

**Table 1:** Summary of devices for determination of thermal conductivity (from [34]).

Method	Temperature Range (K)	Thermal Conductivity Range (W/mK)	Material	Reference
Guarded Hot Plate	80–800	0.001–0.5	Polymers	ASTM C177 Flynn et al. (2002) [35]
Heat Flow Meter	80–800	5–400	Solids	ASTM C518 [36]
Radial Heat Flow	4–1000	>0.1	Solids, Powders	Maglic et al. (1992) [37]
Panel Test	300–1250	0.05–15	Solids	Daryabeigi (1999) [38]
Laser Flash	80–2800	(*)	Solids	ASTM E1461 [39]
Hot Wire	300–1800	<15	Solids, Liquids	ASTM C1113 Roder et al. (2000) [40]

(*) LFA measures thermal diffusivity, but it is able to retrieve the thermal conductivity through temperature-dependent heat capacity and density data.

## Data Availability

The data presented in this study are available on request from the corresponding author.
